# Median effective dose of remimazolam combined with sufentanil for inhibiting laryngeal mask airway insertion responses in children of different ages

**DOI:** 10.3389/fphar.2024.1506209

**Published:** 2025-01-06

**Authors:** Hongyun Li, Jinxia Wang, Rong Wei, Yan Jiang

**Affiliations:** ^1^ Department of Anesthesiology, Shanghai Children’s Hospital, School of Medicine, Shanghai Jiao Tong University, Shanghai, China; ^2^ Department of Clinical research center, Shanghai Children’s Hospital, School of Medicine, Shanghai Jiao Tong University, Shanghai, China

**Keywords:** remimazolam, median effective dose, children, laryngeal mask anesthesia, dixon’s up-and-down method

## Abstract

**Introduction:**

We determined the median effective dose and 95% confidence interval (CI) of remimazolam required to inhibit laryngeal mask airway (LMA) insertion reactions combined with sufentanil 0.3 μg/kg in pediatric anesthesia.

**Methods:**

Children scheduled to undergo elective laryngeal mask anesthesia were divided into the preschool (age: 3–6 years) and school-age (6–12 years) groups. The timer was started after intravenous remimazolam was administered; thereafter, 0.3 μg/kg sufentanil was injected intravenously. The laryngeal mask was placed 3 min after remimazolam was finished. If a positive response to LMA insertion, such as movement, swallowing, coughing, hiccups, or other reactions, was observed during the insertion, the dose was increased by 0.03 mg/kg for the next patient; if there was no response, the dose was decreased by 0.03 mg/kg instead. The trial officially commenced after the first LMA was successfully inserted and continued until alternating positive and negative responses formed seven crossover points. Thereafter, probit regression was performed to calculate the median effective dose (ED_50_) and 95% effective dose (ED_95_) with the corresponding 95% CIs. The time from remimazolam administration to the disappearance of the eyelash reflex was recorded. Heart rate and mean arterial pressure were recorded before (T1, baseline values) and 3 min after (T2) intravenous remimazolam administration. Adverse reactions were also noted.

**Results:**

Overall, 52 children were included; 25 belonged to the preschool group and 27 to the school-age group. In the preschool group, the ED_50_ and ED_95_ for remimazolam and their 95% CIs were 0.476 (0.447–0.517) mg/kg and 0.554 (0.515–0.688) mg/kg, respectively. In the school-age group, the ED_50_ and ED_95_ for remimazolam and corresponding 95% CIs were 0.427 (0.399–0.463) mg/kg and 0.504 (0.467–0.635) mg/kg, respectively. The dosage for the preschool group was significantly higher than that for the school-age group (*p = 0.003*). Conversely, the time from remimazolam administration to the disappearance of the eyelash reflex; LMA insertion success rate; or incidence of coughing, movement, swallowing, and hiccups did not differ significantly between the two groups.

**Conclusion:**

Remimazolam can be safely used for laryngeal mask anesthesia induction in pediatric patients.

**Clinical Trial Registration:**

https://www.chictr.org.cn/, identifier ChiCTR2400087333.

## 1 Introduction

With the development of new anesthetics and the introduction of minimally invasive surgery, laryngeal mask anesthesia is widely applied in short surgical procedures for children (Nevešćanin et al., 2020). Successful laryngeal mask airway (LMA) insertion requires sufficient sedation and analgesia to prevent swallowing, movement, and laryngospasm (Yu et al., 2006). Remimazolam is a novel benzodiazepine derivative that combines the pharmacodynamics of midazolam with a remifentanil-like pharmacokinetic profile; remimazolam has a rapid onset of action and promotes rapid sedation induction (Kilpatrick, 2021). Although the efficacy and safety of remimazolam have been extensively studied in adults (Hirano et al., 2023), current research in children focuses on the determination of effective doses of remimazolam for safety and efficacy and preoperative sedation (Ni et al., 2024; Tobias, 2024). Research on its effective dose for suppressing responses to laryngeal mask placement in children is lacking. Therefore, in this study, a modified Dixon’s up-and-down method was employed to investigate the median effective dose (ED_50_) and 95% effective dose (ED_95_) of remimazolam required to inhibit LMA insertion responses when combined with sufentanil in children. In this study, we aimed to provide novel options for anesthetic administration in pediatric laryngeal mask anesthesia and offer evidence-based references for the rational use of remimazolam.

## 2 Materials and methods

### 2.1 Study design and ethics approval

This study was approved by the Ethics Committee of Shanghai Children’s Hospital (Approval No: 2024R060) and registered in the Chinese Clinical Trial Registry (Registration No: ChiCTR2400087333). Written informed consent was obtained from the parents/guardians of all patients. All procedures adhered to the Declaration of Helsinki. Children scheduled to undergo elective laryngeal mask anesthesia were divided into the preschool (age: 3–6 years) and school-age (age: 6–12 years) groups.

#### 2.1.1 Inclusion criteria

The criteria for enrollment in this study were as follows: age between 3 and 12 years; American Society of Anesthesiologists physical status I or II; body mass index and weight within the normal range for the child’s age; and plan to undergo elective surgery under general anesthesia using a laryngeal mask at Shanghai Children’s Hospital.

#### 2.1.2 Exclusion criteria

The exclusion criteria were as follows: preoperative use of anticonvulsants, sedatives, or medications for attention deficit disorders; pre-existing liver or kidney dysfunction, or other systemic complications; central nervous, respiratory, or circulatory system diseases; psychiatric disorders; and refusal to provide written informed consent either by the child or his/her parents.

### 2.2 Anesthesia method

In this study, no premedication was administered to the children. Participants routinely fast for 6 h and abstain from liquids for 2 h before surgery. Upon entering the operating room, routine electrocardiography, oxygen saturation (SPO_2_), and blood pressure were monitored. Both groups received medication based on the modified Dixon’s up-and-down method. Timing began after intravenous injection of remimazolam, then sufentanil 0.3 μg/kg (administered over 15–30 s) was injected intravenously while the timer was running. Three minutes after the administration of remimazolam, the laryngeal mask was inserted. During this process, if the SPO_2_ dropped to ≤90%, manual ventilation was performed through a face mask. If a positive response to LMA insertion was observed, an additional dose of propofol (1–2 mg/kg) was administered. All anesthesia procedures were performed by the same senior anesthesiologist.

The remimazolam used in this study was provided by Jiangsu Hengrui Pharmaceuticals Co., Ltd (Remimazolam Tosilate for Injection, Specification: 25 mg; Batch No: 231123AK; National Drug Approval No: H20217078). Prior to administration, 25 mL of saline is added to 25 mg of remimazolam to achieve a concentration of 1 mg/mL.

### 2.3 Trial using Dixon’s up-and-down method

A modified Dixon’s up-and-down method was employed (Yin et al., 2017), the initial dose of remimazolam was set at 0.30 mg/kg according to the pretrial results and related studies. If a positive response to LMA insertion was observed, the remimazolam dose for the next child was increased by 0.03 mg/kg. In contrast, the dose was decreased by 0.03 mg/kg for the next patient if a negative response was observed. The trial concluded after the alternating positive and negative responses formed seven crossover points.

### 2.4 Criteria for positive response to LMA insertion

A positive response was defined as the occurrence of reactions that interfered with the quality of the LMA insertion procedure, such as movement, swallowing, coughing, or hiccups.

### 2.5 Rescue measures

If a positive response to LMA insertion was observed, an additional dose of 1–2 mg/kg of propofol was administered. When the SPO_2_ dropped to ≤90%, manual ventilation with a face mask was initiated. If the SPO_2_ continued to decline despite these interventions, the condition was classified as respiratory depression, and emergency endotracheal intubation and mechanical ventilation were performed. Bradycardia was defined as a heart rate (HR) ≤50 beats/min, upon which intravenous atropine 0.01 mg/kg was administered. Hypotension was defined as a ≥ 30% drop in the mean arterial pressure (MAP) from baseline that did not improve within 1 min. Ephedrine was administered in the event of hypotension. Alternatively, a ≥ 30% increase in the MAP from baseline was managed by increasing the depth of anesthesia (intravenous propofol or inhaled sevoflurane). If the blood pressure remained elevated after the intervention, the condition was classified as hypertension. Rescue medications, including atropine (0.01 mg/kg) and epinephrine (0.01 mg/kg), were prepared for all patients throughout the peri-anesthesia period.

### 2.6 Outcome measures

#### 2.6.1 Primary outcome

The primary outcome was the ED_50_ of remimazolam required to inhibit LMA insertion responses in children of different ages and the corresponding 95% confidence interval (CI), which were determined using the Dixon’s up-and-down method.

#### 2.6.2 Secondary outcomes

The secondary outcomes comprised the success rate of LMA insertion; time from remimazolam administration to the disappearance of the eyelash reflex; number of cases where the SPO_2_ dropped to ≤90%; preoperative HR and MAP (T_1_, baseline values); HR and MAP during LMA insertion (3 min after administration of remimazolam, T_2_); and adverse events, including body movement, swallowing, coughing, hiccups, hypotension, hypertension, bradycardia, and respiratory depression.

### 2.7 Statistical analysis

All data were analyzed using IBM SPSS Statistics for Windows, version 26.0 (IBM Corp., Armonk, NY, United States). The Shapiro–Wilk test was used to assess the normality of continuous variables. Normally distributed variables are expressed as the mean ± standard deviation (χ ± s) and were compared using the *t-*test. Non-normally distributed data are expressed as the median (interquartile range) [M (IQR)] and were compared using the non-parametric rank-sum test. Categorical data are presented as frequencies (%) and were analyzed using the chi-squared test or Fisher’s exact test. Paired *t*-tests or non-parametric rank-sum tests were employed to evaluate the differences in hemodynamic parameters between T_1_ and T_2_. Probit regression was performed to calculate the ED_50_ and the corresponding 95% CI of remimazolam for inhibiting the LMA insertion response in children when combined with sufentanil. Dixon’s up-and-down plots and dose–response curves were generated using GraphPad Prism 9 (GraphPad, San Diego, CA, United States). *p*-values < 0.05 were considered statistically significant.

## 3 Results

### 3.1 Patient characteristics

**FIGURE 1:**
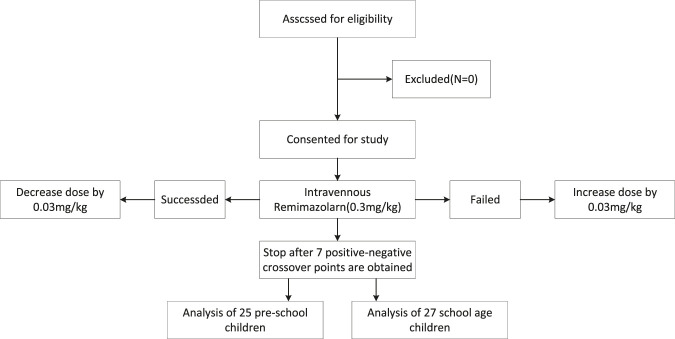
A total of 52 children were included in the study at the completion of 7 turning points; 25 children belonged to the preschool group and 27 to the school-age group.

A total of 52 children were included in the study at the completion of 7 turning points; 25 children belonged to the preschool group and 27 to the school-age group as shown in Figure 1. The general characteristics of the two groups are presented in Table 1.

**TABLE 1:** General characteristics of children in both groups.

Variable		Preschool group (n = 25)	School-age group (n = 27)
Sex (N/%)	M	18 (72)	23 (85.19)
	F	7 (28)	4 (14.81)
Age (years)		4 (3, 5)*	7.58 (6.5, 9.75)
Height (cm)		107.5 (103, 117)*	129 (120, 142)
Weight (kg)		19 (16, 23.1)*	26 (22.2, 38)
BMI (kg/m^2^)		15.37 ± 1.48*	16.67 ± 2.54

BMI: body mass index,*p < 0.05.

### 3.2 Primary outcomes

**FIGURE 2:**
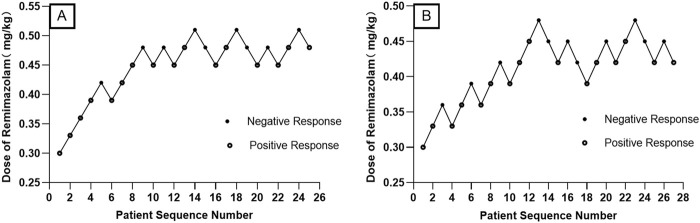
Modified Dixon’s up-and-down plots for the remimazolam dose required to inhibit LMA insertion responses in preschool children **(A)** and school-aged children **(B)** LMA: laryngeal mask airway.

**FIGURE 3:**
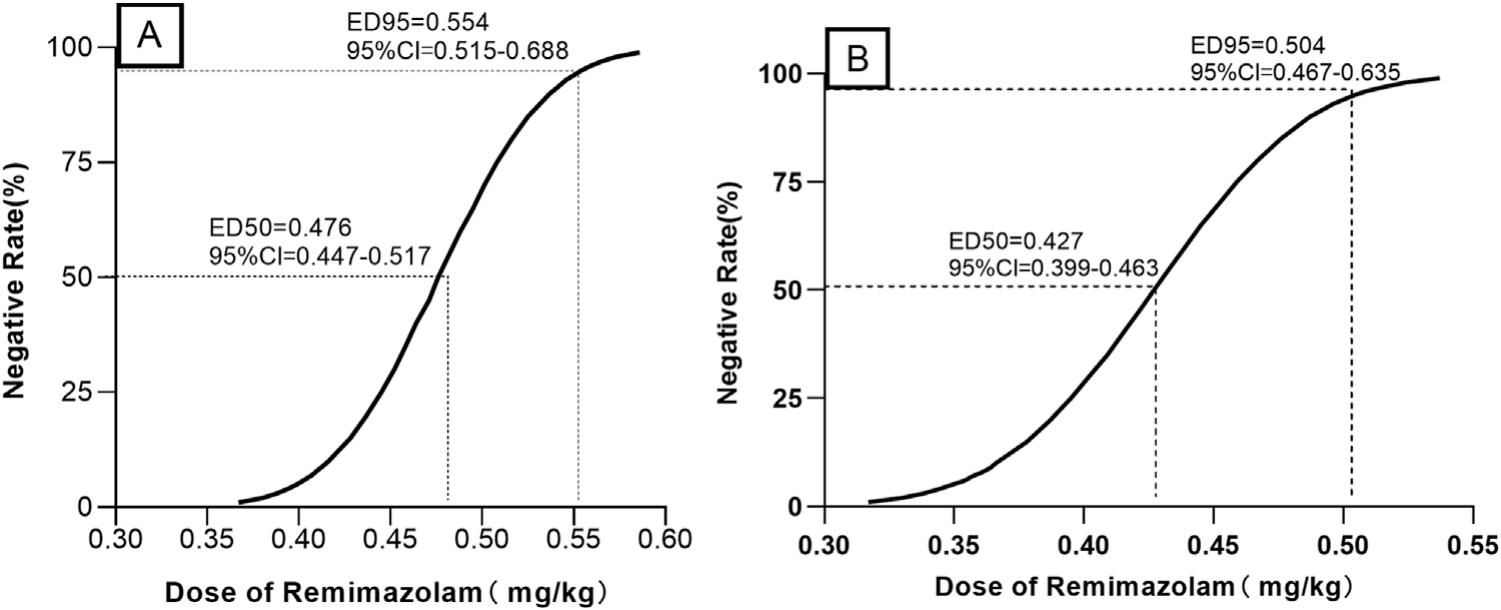
Dose–response curves for remimazolam dose required to inhibit LMA insertion responses in preschool children **(A)** and school-aged children **(B)** LMA: laryngeal mask airway.

When combined with sufentanil 0.3 μg/kg, the ED_50_ (95% CIs) and ED_95_ (95% Cis) for remimazolam required to inhibit LMA insertion responses were 0.476 (0.447–0.517) mg/kg and 0.554 (0.515–0.688) mg/kg for preschool children, respectively. In contrast, for school-age children, they were 0.427 (0.399–0.463) mg/kg and 0.504 (0.467–0.635) mg/kg, respectively. The modified Dixon’s up-and-down plots are illustrated in Figures 2A, B. The dose-response curves of remimazolam for inhibiting a LMA insertion response plotted using the results of the probability analysis are depicted in Figures 3A, B.

### 3.3 Secondary outcomes

#### 3.3.1 LMA insertion outcomes

None of the children in this study experienced respiratory depression. LMA insertion was successful in 9 of 25 children in the preschool group, while 16 required rescue medication. The laryngeal mask was successfully inserted in 11 of 27 children in the school-age group, while 16 required rescue medication. No significant difference was observed in the success rates of LMA insertion between the two groups (*p* = 0.726) (Table 2).

**TABLE 2:** Incidence of adverse reactions in both groups.

Variable	Preschool group (n = 25)	School-age group (n = 27)
Remimazolam dose (mg/kg)	0.45 (0.42, 0.48)*	0.42 (0.39, 0.45)
Time to the disappearance of eyelash reflex (seconds)	48 (40, 62)	40 (35, 52)
LMA insertion success rate, yes, n (%)	9 (36)	11 (40.74)
SpO_2_ ≤ 90%, yes, n (%)	19 (76)	22 (81.48)
Body movement, yes, n (%)	4 (16)	10 (37.04)
Swallowing, yes, n (%)	13 (52)	8 (29.63)
Coughing, yes, n (%)	5 (20)	2 (7.41)
Hiccups, yes, n (%)	4 (16)	3 (11.11)
Injection Pain, yes, n (%)	—	1 (3.7)

LMA, laryngeal mask airway; SPO_2_, oxygen saturation,*p < 0.05.

#### 3.3.2 Time from remimazolam administration to the disappearance of the eyelash reflex

The time from the start of sufentanil injection until the eyelash reflex disappeared (defined as the end of remimazolam injection) was 48 (40, 62) s in the preschool group and 40 (35, 52) s in the school-age group, and the difference between the two groups was not statistically significant (Table 2).

#### 3.3.3 Positive response during LMA insertion

The details of positive responses elicited during LMA insertion are depicted in Table 2.

#### 3.3.4 Hemodynamic changes

##### 3.3.4.1 MAP change

After anesthesia induction, the MAP declined in both groups compared to the pre-induction levels. At both T_1_ and T_2_, the MAP of preschool children was lower than that of school-aged children (Table 3). Four (16%) children in the preschool group and three (11.11%) in the school-age group experienced a ≥ 20% change in the MAP. One child each in the preschool group (4%) and school-age group (3.7%) experienced a ≥ 30% change in the MAP.

**TABLE 3:** Changes in the MAP/HR in both groups.

Variable	Preschool group (n = 25)	School-age group (n = 27)
MAP (T1) (mmHg)	74.4 (67.25, 83.75)^a^ ^,^ ^b^	82 (76, 86)^b^
MAP (T2) (mmHg)	67.5 (63.25, 75.75)^a^	72 (66, 83)
HR (T1) (bpm)	94 (87, 114.75)	92 (82.25, 105.5)
HR (T2) (bpm)	102 (97, 112)^a^	94 (87.5, 101.75)

MAP, mean arterial pressure; HR, heart rate. T_1_, baseline values; T_2_, 3 min post-administration.

^a^ Comparison between the two groups.

^b^ Comparison between T1 and T2 values, *p* < 0.05.

##### 3.3.4.2 HR change

None of the children in this study experienced bradycardia. At T_1_, there was no significant difference in the HR between the two groups (94 vs. 92 beats/min). However, at T_2_, the HR of preschool children was higher than that of school-age children (102 vs. 94 beats/min) (Table 3). Six (24%) children in the preschool group and three (11.11%) in the school-age group experienced a ≥ 20% change in the HR. Two (8%) preschool children experienced a ≥ 30% change in the HR.

## 4 Discussion

Co-administering opioids with sedatives is a common practice in laryngeal mask anesthesia. Currently, sufentanil is the most effective available opioid analgesic, which also exhibits minimal cardiovascular effects (Yin et al., 2017). A previous study suggested that sufentanil administered at a dose of 0.3 μg/kg was optimal for controlling cardiovascular responses during the induction of anesthesia in children (Xue et al., 2008). Consequently, the sufentanil dose utilized in this study was 0.3 μg/kg. The modified Dixon’s up-and-down method employed in the study is a well-established approach for calculating the ED_50_, allowing for the estimation of the dose–response relationship of the drug based on the ED_50_ (Oron et al., 2022). The results of the current study indicated that, when combined with 0.3 μg/kg sufentanil, the ED_50_ and ED_95_ of remimazolam for inhibiting LMA insertion responses in preschool children were 0.476 (0.447–0.517) mg/kg and 0.554 (0.515–0.688) mg/kg, respectively. For school-aged children, the ED_50_ and ED_95_ of remimazolam were 0.427 (0.399–0.463) mg/kg and 0.504 (0.467–0.635) mg/kg, respectively. Therefore, the remimazolam dosage required for preschool children was higher than that for school-age children. Based on the allometric theory, Anderson and Holford proposed that compartmental volumes scale linearly with size. Pharmacokinetic studies of remimazolam have shown lower clearance in children than in adults, which may be related to maturation (Anderson and Holford, 2008; Gao et al., 2023).

A study in adults showed that when combined with remifentanil (TCI3.0 ng/mL), the ED_95_ of remimazolam required for successful insertion into the i-gel laryngeal mask is 0.182 mg/kg, which is significantly lower than the dose in our study. This may be due to the difference in opioids used and age of the participants (Cho et al., 2024). Oh’s research showed that the ED_95_ of remimazolam for general anesthesia induction in young adults was 0.367 mg/kg, 0.369 mg/kg in middle-aged adults, and 0.249 mg/kg in older adults (Oh et al., 2022). Another study that included adults showed that upon combination with remifentanil, the ED_50_ and ED_95_ of remimazolam required for successful LMA insertion were 0.244 mg/kg and 0.444 mg/kg, respectively (Oh et al., 2023). These adult doses are significantly lower than those used in this pediatric study. This disparity can be attributed not only to differences in the analgesic agents but also to the pharmacokinetic variations between children and adults. Additionally, different definitions of primary outcome measures can influence the effective dose of remimazolam.

Research has indicated that when mean bispectral index values were within the same range as propofol, remimazolam mitigated the incidence of hypotension (Doi et al., 2020). A study exploring the dosage and safety of remimazolam found that the ED_50_ and ED_95_ for respiratory depression were 0.19 mg/kg and 0.27 mg/kg, respectively (Chae et al., 2022). A total of 255 patients were administered remimazolam combined with alfentanil for sedation during endoscopic retrograde cholangiopancreatography procedures; 9.6% of the patients in the remimazolam group developed hypoxia (Dong et al., 2023). In this study, while 19 (76%) preschool and 22 (81%) school-aged children exhibited an SpO_2_ level of ≤90% prior to LMA insertion (within 180 s of remimazolam injection), the level rose rapidly after supplemental oxygen administration. The significant difference in the incidence of low SpO_2_ between the two studies may be because supplemental oxygen (6 L/min) was administered to the patients by nasal cannula in the previous study and a combined alfentanil dosage of 10 μg/kg, compared to a dose of 0.3 μg/kg used in this study. The higher sufentanil dosage, combined with the synergistic effects of remimazolam, probably increased the incidence of hypoxemia in the absence of supplemental oxygen (Bevans et al., 2017). In this study, both groups exhibited a decrease in the MAP at T_2_ relative to T_1_, while the HR increased in both groups at T_2_ relative to T_1_. These findings align with those of previous studies that reported a reduction in blood pressure and elevation in the HR during remimazolam infusion (Pesic et al., 2020; Choi et al., 2022).

A previous study reported that 18.7% of patients receiving propofol experienced pain at the injection site, while no such pain was observed in patients treated with remimazolam (Sneyd et al., 2022). In this study, one (3.70%) school-aged child experienced injection pain, whose incidence was significantly lower than that associated with propofol. Meanwhile, some preschool children experienced crying and agitation after receiving remimazolam since they did not receive other sedatives prior to the procedure, making it difficult to accurately quantify the incidence of injection pain in this age group.

This study has some limitations. First, the fixed dose of sufentanil may have influenced the effective dosage range of remimazolam. Second, the use of probit regression to derive the ED_95_ from the ED_50_ could have resulted in underestimation of the effective dosage range. Third, the administration of supplemental oxygen via a mask during remimazolam administration could have reduced the incidence of decreased SpO_2_.

To conclude, the combination of remimazolam and sufentanil for LMA insertion in children of various ages is typically successful and safe when administered and monitored correctly. While it has various advantages, including easy insertion, stable hemodynamics, and quick recovery, the danger of respiratory depression demands close postoperative monitoring, particularly in younger children. To achieve the best results, the dose should be tailored to each child’s age and physiological parameters.

## Data Availability

The original contributions presented in the study are included in the article/supplementary material, further inquiries can be directed to the corresponding authors.

## Abbreviations

CI, confidence interval; ED_50_, median effective dose; ED_95,_ 95% effective dose; HR, heart rate; IQR, interquartile range; LMA, laryngeal mask airway; MAP, mean arterial pressure; SPO_2_, oxygen saturation.
